# Flaxseed Gum/Arabic Gum/Tween 80-Based Oleogel as a Fat Substitute Applied in Emulsified Sausage: Physicochemical Properties, Sensory Attributes and Nutritional Quality

**DOI:** 10.3390/gels9090759

**Published:** 2023-09-18

**Authors:** Qiaomei Zhu, Fu Chen, Peiyang Li, Tao Wu, Yijun Pan, Min Zhang

**Affiliations:** 1 State Key Laboratory of Food Nutrition and Safety, College of Food Science and Engineering, Tianjin University of Science and Technology, Tianjin 300457, China; fuchetust@163.com (F.C.); wutao@tust.edu.cn (T.W.); 2 Tianjin Modern Innovative TCM Technology Co., Ltd., Tianjin 300392, China; 3 Department of Food Science, Rutgers, The State University of New Jersey, 65 Dudley Road., New Brunswick, NJ 08901, USA; yp238@scarletmail.rutgers.edu; 4 China-Russia Agricultural Processing Joint Laboratory, Tianjin Agricultural University, Tianjin 300384, China

**Keywords:** flaxseed gum, oleogels, fat replacers, rheological properties, emulsified sausage

## Abstract

In the present study, flaxseed gum (FG), Arabic gum (GA) and Tween 80 were used to prepare oleogels through an emulsion-templated method, and the obtained oleogels were designed for the partial substitution of pork fat in emulsified sausage. An increment in FG concentrations enhanced the viscoelasticity of emulsions, which resulted in the improved stability of emulsion systems, with smaller droplet sizes. In addition, increased FG concentrations contributed to higher mechanical strength, denser network structure and lower oil loss of oleogels. As a fat substitute, the prepared oleogels improved the textural properties and nutritional quality of emulsified sausages. With the increase in the substitution level of oleogels, the hardness and chewiness of the emulsified sausage increased, and the cooking loss decreased. Meanwhile, the reformulation with oleogels decreased the saturated fat from 57.04 g/100 g lipid to 12.05 g/100 g lipid, while increasing the ratio of omega-6 to omega-3 essential fatty acids from 0.10 to 0.39. The obtained results demonstrated that the flaxseed gum/Arabic gum/Tween 80-based oleogels had huge potential to successfully replace pork fat in emulsified sausage products.

## 1. Introduction

Emulsified sausages are popular meat products that are widely accepted by consumers in many countries. However, the animal fat contains a high level of saturated fatty acids (SFA) and cholesterol, which is considered to be correlated with health problems such as cardiovascular disease, metabolic syndrome and obesity [1,2]. It has been recommended by the World Health Organization that saturated fat consumption should be reduced to less than 10% of total energy per day [2]. Therefore, the development of healthier emulsified sausages with reduced SFA and cholesterol has become a key challenge for the meat industry to meet the requirements of the commercial market.

Recently, organogelation, a novel structured oil technique that converts the healthy liquid oil to a three-dimensional gel state as a fat replacer, has drawn noticeable attention from researchers. Oleogels can not only mimic the solid-like state of animal fat but also endow food products with the health characteristics of unsaturated fatty acids. Wolfer et al. [3] prepared rice bran wax-based oleogels to substitute pork back fat in frankfurter-type sausages. The obtained results showed that the oleogels were able to match pork back fat in adhesiveness, cohesiveness and chewiness. Another study [4] reported that beeswax-based sesame oil oleogels decreased the hardness, chewiness and gumminess of burgers formulated with animal fat. In addition, the incorporation of oleogels resulted in an 11% reduction in cooking loss and a 1.6% reduction in fat absorption. It has been stated that ethylcellulose-based oleogels could reduce water loss in meat batter and maintain similar textural properties as animal fat [5]. More importantly, the oleogels can modify the fatty acid compositions of meat products. A more recent study by Ferro et al. [6] reported that the technological properties of oleogel-formulated Bologna sausages were not affected by the presence of high-oleic sunflower oil, and its unsaturated fatty acid contents, especially the oleic acid and linoleic acid, were significantly increased. All these studies reveal the huge potential of oleogels in replacing the animal fat of processed meat products to enrich their nutritional quality.

Generally, lipid-based oleogelators such as varied plant wax, fatty acid derivatives, oryzanol and phystosterols are directly used to fabricate oleogels. These oleogelators can trigger oil gelation via crystallization or self-assembly behavior. Apart from the direct method, indirect oleogelation based on the emulsion template approach with food-approved hydrocolloids tends to be more promising as a result of the low cost, massive production and commercial availability of hydrocolloids [7,8]. The emulsion template method involves the preparation of oil-in-water (O/W) emulsion followed by removing water molecules to obtain dried oleogel samples. Due to the high molecular weight of biopolymers, to induce the formation of a gel network, the usage amount of structuring agents is remarkably low [1]. Hydrocolloids such as ethyl cellulose and hydroxypropyl methylcellulose (HPMC) are commonly used to fabricate oleogels, since they are amphiphilic with certain surface-active properties [9,10]. Some polysaccharides have no tendency for oil absorption, and they are generally used in combination with other structuring agents or emulsifiers to increase the oil-binding capacity. Espert et al. [11] prepared oleogels with xanthan gum as a thickening agent in combination with different structuring agents such as locust bean gum, Tween 80, whey protein and soy lecithin, and all prepared oleogels showed a high oil-binding capacity.

In the present study, flaxseed gum, Arabic gum and Tween 80 were used as structuring agents to fabricate oleogels. Flaxseed gum (FG) is extracted from flaxseed and its hull, and it has excellent water-holding capacity, rheological properties and gelling and emulsifying capacity [12]. Arabic gum (GA) is widely used as an emulsifier in the food industry due to its excellent emulsifying properties. The stability of an emulsion system during the drying process is important for maintaining the oil-binding capacity of oleogels. The presence of FG and GA could act as both an emulsifier and a thickener to enhance the stability of the emulsion system, which might efficiently prevent oil leakage during the freeze-drying of emulsion droplets. In addition, Tween 80 was used as an emulsifier to obtain O/W emulsions. It has been reported that Tween 80 could promote the gelation of Span80-based oleogels [13]. The combination of Tween 80 and GA with FG might effectively form the oleogels at a lower concentration. Afterwards, the impact of the substitution ratio of oleogels on the textural properties, microstructure and nutritional properties of emulsified sausages is investigated, in order to explore the potential application of the obtained oleogels in the fat replacement of meat products.

## 2. Results and Discussion

### 2.1. Particle Size and Microstructure of O/W Emulsions

**Figure 1:**
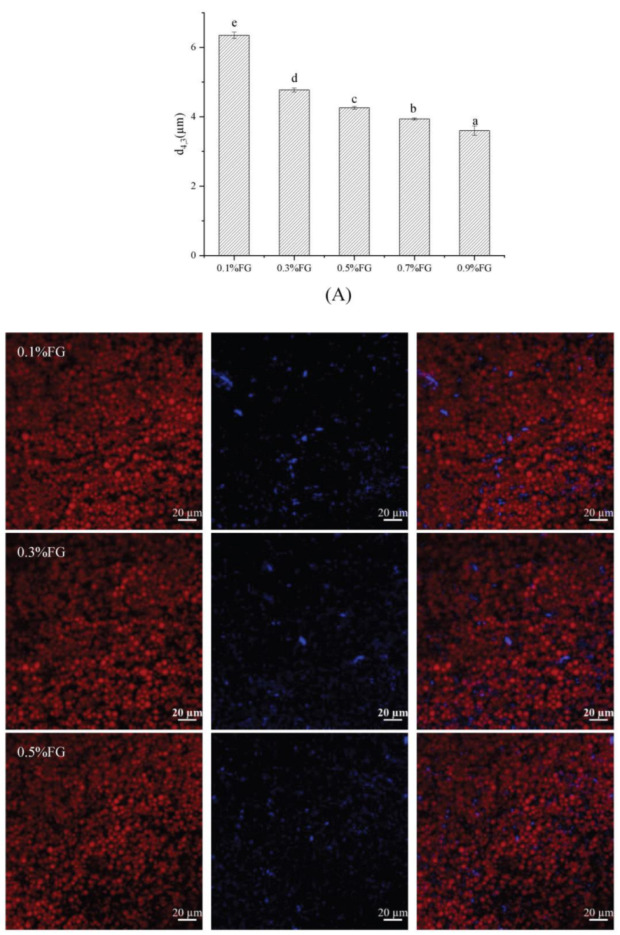

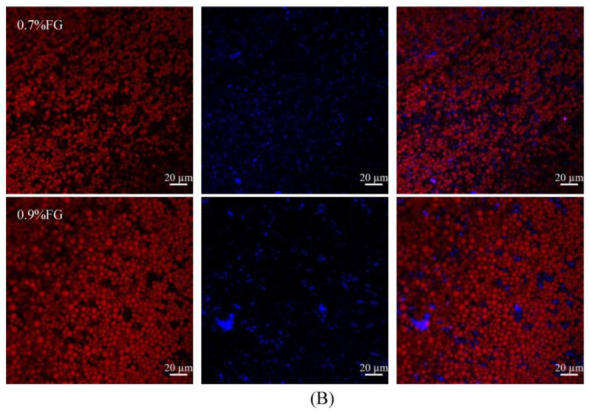
The average particle size (**A**) and micrographs of emulsions with FG concentration of 0.1, 0.3, 0.5, 0.7 and 0.9 wt% under confocal laser scanning microscopy (CLSM) (**B**). The lowercase letters in (**A**) indicated the significance between different treatment control.

The impact of FG concentrations on the particle size and microstructure of emulsions was investigated, as shown in Figure 1. FG concentrations significantly affect the average particle size of O/W emulsions stabilized by Tween 80 and Arabic gum (GA). With an increasing FG concentration from 0.1 wt.% to 0.9 wt.%, the particle size significantly decreased from 6.4 μm to 3.6 μm. The reason for this was attributed to the sufficient adsorption of FG onto the oil–water interface, which favored the formation of smaller droplets. This was in consistency with the result of a previous study by Sun et al. [14] that higher concentrations of FG could efficiently wrap the surface of oil droplets, thereby producing smaller emulsion droplets during homogenization. Figure 1B exhibits a three-dimensional network of aggregated droplets, and the results confirmed that the emulsions’ droplet size tended to be smaller with increased FG concentrations. The formation of aggregates might be attributed to an attractive interaction of droplets. A previous study by Liu et al. [15] reported that higher FG concentrations could cause the bridging flocculation of emulsion droplets. Moreover, the purple color indicates the presence of FG, suggesting that except for adsorption onto the oil–water interface, excess FG molecules were present in the continuous aqueous phase. The dispersing FG would further increase the viscosity of the emulsion and enhance the system’s stability.

### 2.2. Rheological Characterization of Emulsions and Oleogels

**Figure 2:**
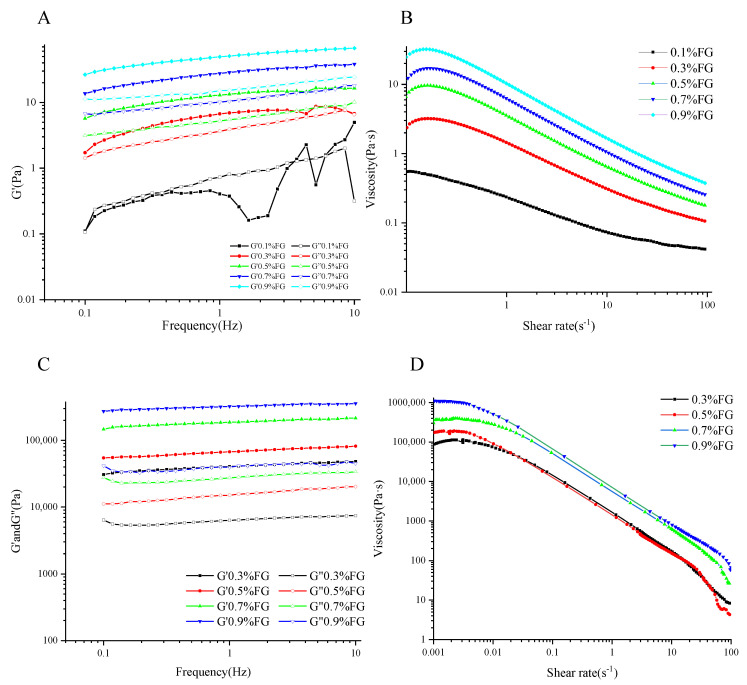
Frequency sweep (**A**) and flow curve (**B**) of emulsions with different FG concentrations. Frequency sweep (**C**) and flow curve (**D**) of oleogels with different FG concentrations.

The rheological properties of the emulsion and oleogels with varied concentrations of FG were evaluated by flow measurement frequency sweep, as shown in Figure 2. The apparent viscosity of emulsions showed a downward trend with shear rates, indicating a shear-thinning behavior. Within the shearing rate range (0.01–100 s^−1^), the increase in FG concentrations resulted in higher viscosity of the emulsion system. As described above, the presence of unadsorbed FG molecules in the continuous phase could enhance the viscosity of emulsions. Higher viscosity is beneficial for reducing the mobility and collision frequency of droplets, thus improving the stability of emulsions against coalescence [16]. Within the frequency range (0.1–10 Hz), the elastic modulus (G′) was higher than the viscosity modulus (G″), indicating the existence of a dominant solid-like gel structure [12]. A slight frequency dependence could be observed for both the G′ and G″, suggesting a typical weak gel behavior [17]. The increase in FG concentrations led to a higher viscoelastic modulus of emulsions, reflecting a stronger gel structure.

The rheological properties of oleogels were explored, as shown in Figure 2C,D. After freeze-drying, the oleogel prepared with 0.1 wt.% FG was not stable, and it is not discussed in the following study. The gel strength of oleogels was significantly affected by FG concentrations. At a fixed frequency of 0.1 Hz, when the FG concentration increased from 0.3 wt.% to 0.9 wt.%, the G′ significantly increased from 30,752.9 Pa to 273,514.2 Pa. The reason for this might be that a higher FG concentration led to the formation of a tighter network of oleogels, thus increasing their mechanical strength. A similar trend was also observed by Meng et al. [8] and Espert et al. [11]. The G′ and G″ were independent of frequency, indicating a strong gel structure of oleogels. The oleogels also exhibited a shear-thinning behavior, as shown in Figure 2D, and higher FG concentrations increased the apparent viscosity.

### 2.3. Microstructure and Oil Loss of Oleogels

**Figure 3:**
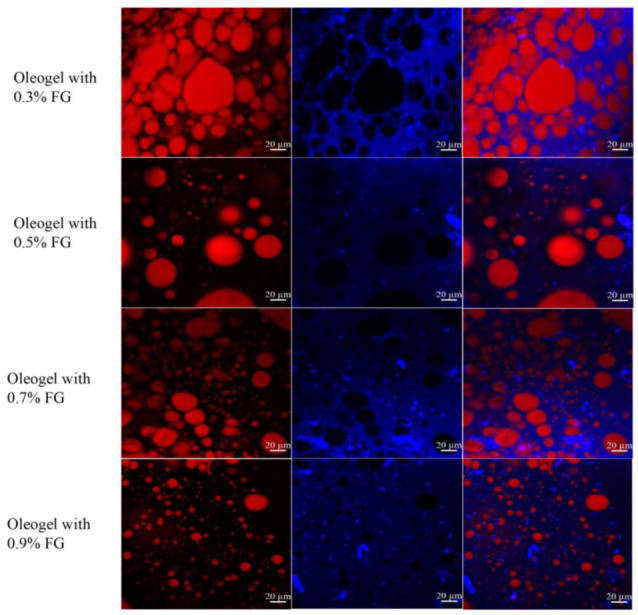
CLSM images of oleogels with FG concentrations varying from 0.3 wt.% to 0.9 wt.%.

The microstructure of oleogels was observed by confocal laser scanning microscopy (CLSM). The red and blue color present the oil phase and polysaccharide regions, respectively, as shown in Figure 3. The oil droplets were dispersed throughout the polysaccharide network, indicating that the freeze-drying treatment did not completely destroy the emulsion droplets. However, compared with the original O/W emulsions, the droplets within the oleogels were much larger, suggesting that coalescence occurred during the freeze-drying process. With increasing FG concentrations, the entrapped emulsion droplets tended to be smaller, which is a similar trend as original O/W emulsions. A higher FG concentration could decrease the coalescence rate of emulsion droplets. Meanwhile, the polysaccharide gel network became more compact, and this was beneficial for the entrapment of oil in the porous gel structure.

**Figure 4:**
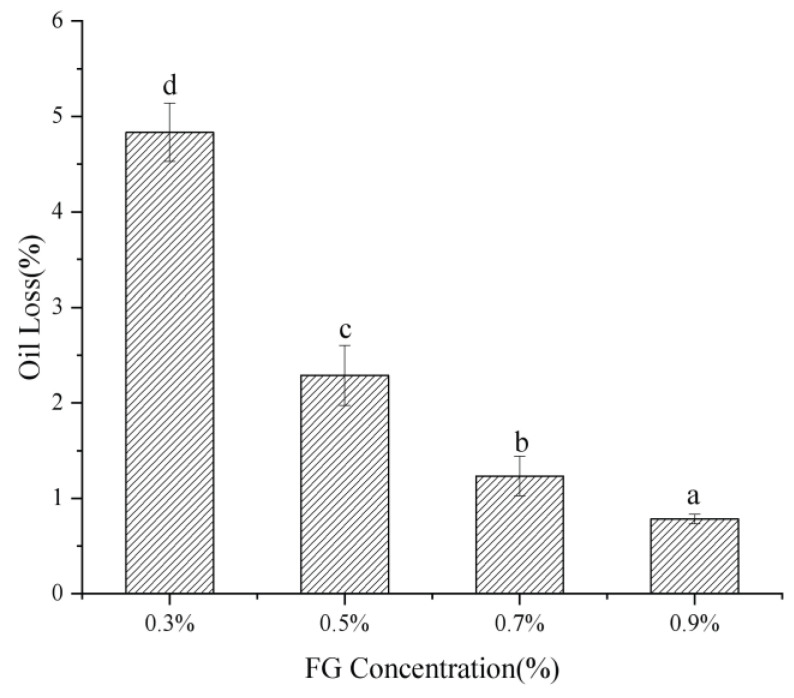
Oil loss of oleogels with varied FG concentrations. The lowercase indicated the significance between samples.

Oil loss is an important factor gauging the ability of an oleogel to retain oil and maintain structural stability. The oil loss of oleogels prepared with varied FG concentrations is shown in Figure 4. Increasing the FG concentrations could significantly decrease the oil loss rate. The oil loss of oleogels with 0.3 wt.% FG was 4.83%, and it decreased to 0.78% when the FG concentration reached 0.9 wt.%. The decrease in oil loss was due to the more compact network structure of the oleogels.

### 2.4. Chemical Characterization by ATR-FTIR Spectroscopy

**Figure 5:**
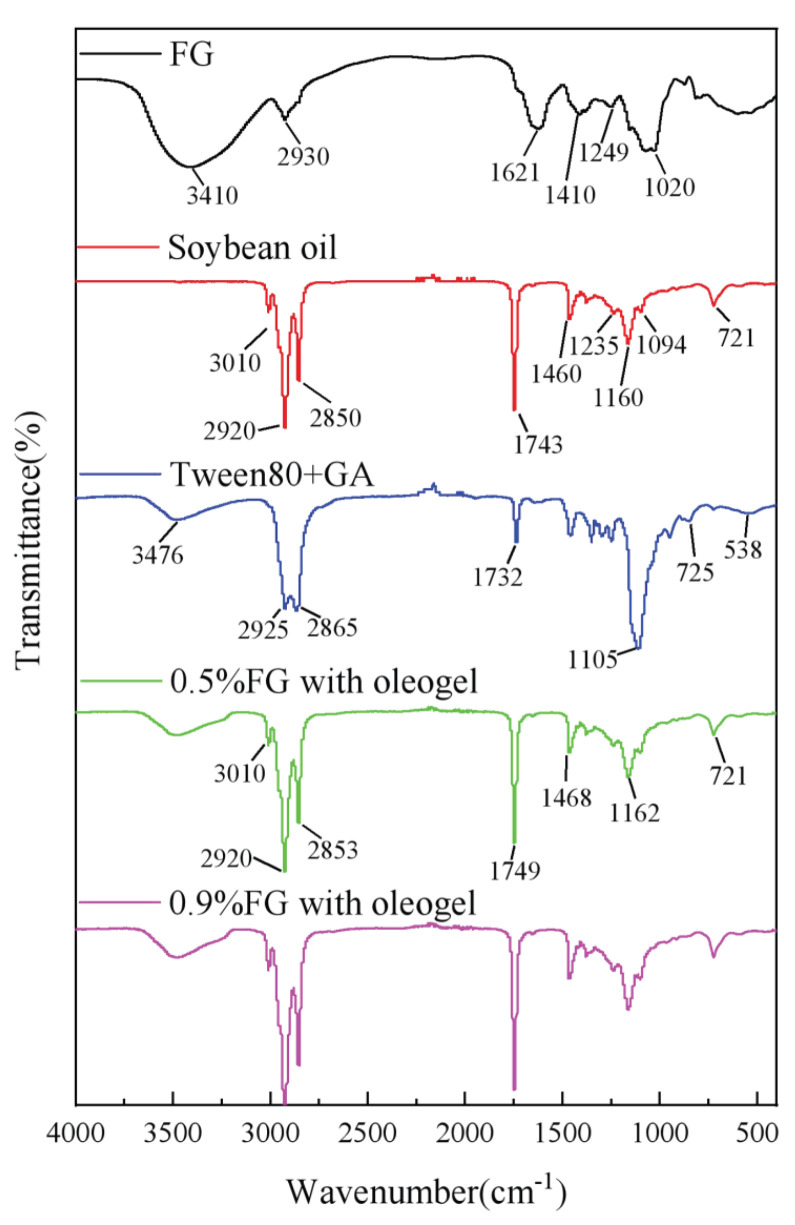
FTIR spectra of pure soybean oil, FG powers, Tween 80/Arabic gum mixture powders and oleogels.

The molecular structure of oleogels was analyzed by FTIR, and the spectra of pure FG powder, a mixture of GA/Tween 80, soybean oil and oleogels are shown in Figure 5. The adsorption bands of the oleogels are associated with the functional groups in soybean oil and the mixture of FG/GA/Tween 80. An adsorption peak at around 3400 cm^−1^ could be observed in spectra of the oleogel, which is related to the stretching vibration of -OH groups [18]. Meanwhile, adsorption peaks at 3410 and 3476 cm^−1^ were also observed for the pure FG and the mixture of GA/Tween samples, respectively, suggesting that the intramolecular or intermolecular hydrogen bonds present in oleogels came from the polysaccharides. A small sharp peak was observed in oleogels at 3010 cm^−1^, which is due to the C-H stretching vibration in =C-H. The adsorption band at 2920 and 2850 cm^−1^ in pure soybean oil and that at 2925 and 2865 cm^−1^ in the mixture of GA/Tween 80 indicate the C-H stretching vibrations of CH_2_ and CH_3_ [8]. These adsorption bands shifted to 2920 and 2853 cm^−1^ in oleogels, which might be attributed to the organization of alkyl groups via the van der Waals interaction [19]. The adsorption band at 1732 cm^−1^ in soybean oil was linked to C=O stretching bonds, and it shifted to 1749 cm^−1^ for the oleogels. Peaks observed at 1468 cm^−1^ and 1162 cm^−1^ correspond to the C-H bending vibration of CH_3_ and the C-O stretching vibration of C-O-C, which is linked to the soybean oil and polysaccharides. Peaks at 721 cm^−1^ observed in both soybean oil and oleogels represent the bending vibration of (CH_2_)_n_, as reported by Ma et al. [20]. There was no significant difference between the oleogels with varied concentrations of FG. It was speculated that the hydrogen bonding present in the polysaccharides FG and GA was the main driving force for the formation of oleogels.

### 2.5. Characterization of Emulsified Sausage

#### 2.5.1. Proximate Composition and Color

Oleogels with 0.7 wt.% FG were used as a fat substitute for emulsified sausages. The proximate compositions and color of sausages formulated with pork back fat and oleogels are shown in Table 1. The moisture content of the control sausage was 60.15%, and it increased from 60.73% to 63.24% in sausage samples with an increasing substitution level of oleogel from 25% to 100%. The increment in the moisture content of emulsified sausages was attributed to the presence of a higher amount of water in oleogels prepared with polysaccharides, as reported by Fontes-Candia et al. [21]. It should be noticed that the substitution of pork fat by oleogels caused a significant decrease in fat content, from 28.53% to 1.52%. These results are in agreement with previous study by da Siva et al. [22]. Meanwhile, the presence of oleogels resulted in a slight increase in the protein content of emulsified sausages, and this could be attributed to the presence of protein in FG and GA. 

Color parameters are an important factor gauging the sensory quality of emulsified sausages. As shown in Table 1, the L*, a* and b* values were significantly affected by the fat reformulations. Compared with the control group, the whiteness (L*) value of emulsified sausages formulated with oleogels tended to increase. This result is in agreement with Ferro et al. [6], who reported that the replacement of back fat with oleogels could increase the whiteness of Bologna sausages. The fortified whiteness of emulsified sausage might be attributed to the smaller emulsion droplets within oleogels, which could reflect more light [23]. The parameters of a* and b* indicate the red and yellow color of emulsified sausages. The replacement of back fat with oleogels resulted in a decrease in a* but an increase in the b* value of sausages. The soybean oil is yellow in color, and therefore, it caused an increase in the yellowness of emulsified sausages.

#### 2.5.2. Texture Profile Analysis (TPA)

**Figure 6:**
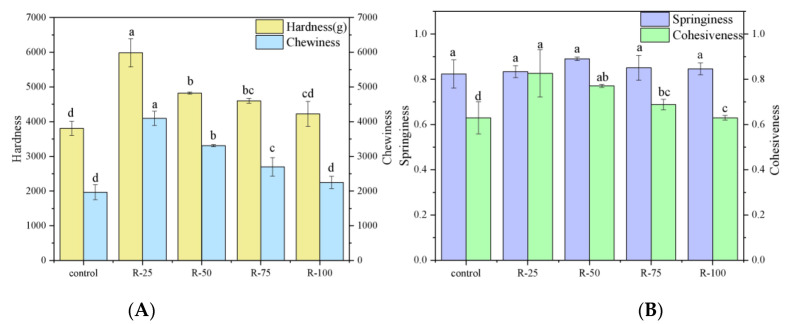
Textural profiles of emulsified sausages with varied fat substitution by oleogels: hardness and chewiness (**A**), springiness and cohesiveness (**B**). The lowercase letters indicated the significance between samples.

The textural characteristics (hardness, chewiness, springiness, cohesiveness) of emulsified sausages formulated with a varied substitution ratio of pork back fat by oleogels were investigated, as shown in Figure 6. Compared with the control group, the hardness values of the sausages formulated with oleogels were higher. This result might be associated with the stronger mechanical properties of oleogels as compared to the pork back fat. When the substitution ratio of oleogels increased from 25% to 100%, the hardness of the emulsified sausages decreased. The hardness of emulsified sausages formulated with 100% oleogels was similar to that of the control group. The cohesiveness and chewiness of sausages showed a similar trend as hardness. However, there was no significant difference in springiness between the control batch and the emulsified sausages with oleogels. A similar trend has been reported by previous studies when using oleogels to replace pork back fat in Bologna sausages and burgers [22,24]. 

#### 2.5.3. Water-Holding Capacity, Cooking Loss and Microstructure of Emulsified Sausages

**Figure 7:**
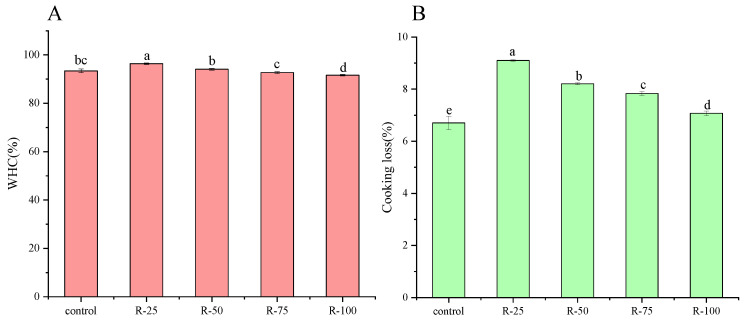
WHC (**A**) and cooking loss (**B**) of emulsified sausage with different fat substitution. The lowercase letters indicated the significance between samples.

The water-holding capacity and cooking loss of varied emulsified sausages are shown in Figure 7. The WHC of the control group was 93.40%, which was lower than that of the R-25 and R-50 samples. It was reported that higher WHC was beneficial for retaining water molecules within the gel structure and reducing the cooking loss of sausages [25]. The presence of flaxseed gum and Arabic gum in oleogels was most likely to increase the water-binding capacity of sausages. However, further increasing the substitution level of oleogels, the WHC decreased from 92.70% to 91.60%. This might be attributed to the weakening interaction between the oleogels and myofibrillar protein, which led to a decreased structural integrity of the final sausage products and reduced the WHC. The fat substitution by oleogels significantly affected the cooking loss of the emulsified sausages. As shown in Figure 7B, the cooking loss of the emulsified sausages formulated with oleogels varied from 9.10% to 7.10% with an increased substitution level of oleogels, which was higher than that of the control group, with a value of 6.70%.

**Figure 8:**
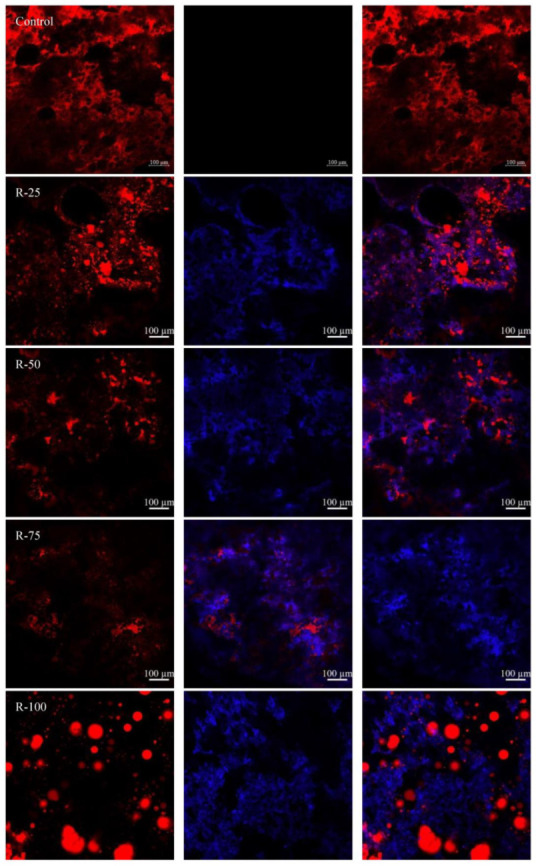
CLSM images of emulsified sausage.

The CLSM images of emulsified sausages with different substitution ratios of oleogels are present in Figure 8. The red color and blue color indicate the distribution of oil and polysaccharide gel in the sausages. It was obvious that with increased fractions of oleogels within the sausages, the regions of the oil phase indicated by red color are reduced, suggesting that the fat contents decreased. This was consistent with the fat content result in Table 1. When the substitution level was lower than 75%, the emulsion droplets were disrupted during the preparation of the emulsified sausages. Meanwhile, partially flocculated and relatively large emulsion droplets could be still observed in R-100. The emulsion droplets were trapped in the three-dimensional network, which is beneficial for reducing the water loss.

#### 2.5.4. Fatty Acid Profiles of Emulsified Sausages

The effect of the replacement of pork back fat by oleogels on the fatty acid compositions is displayed in Table 2. Palmitic acid (C16:0) and stearic acid (C18:0) are the main saturated fatty acids (SFA) in emulsified sausage. The stearic acid contents of R75 and R100 samples were lower than that of the control group. The SFA contents of emulsified sausages formulated with oleogels were significantly lower than that of the control group (57.04 g/100 g lipid). The content of SFA decreased by 78.87% when the pork back fat was completely replaced by oleogels, and this was related to the lower content of SFA in oleogels. Asuming-Bediako et al. [26] also found a similar reduction, and they studied a method of replacing pig back fat with HOSO in sausages. Therefore, replacing fat with oleogels can reduce saturated fatty acid content because the SFA is reduced by more than 30%. Oleic acid (C18:1*n*9) and linoleic acid (C18:2*n*6) were the predominant monounsaturated fatty acid (MUFA) and polyunsaturated fatty acid (PUFA) in emulsified sausages formulated with oleogels. With the increase in the substitution ratio of oleogels, the amount of total MUFA and PUFA in sausages became higher. After completely replacing the pork back fat with oleogels, the amount of MUFA and PUFA increased from 5.09 g/100 g lipid to 28.08 g/100 g lipid and from 14.35 g/100 g lipid to 52.68 g/100 g lipid, respectively. The lipid composition results were consistent with previous studies that reported that oleogels as a fat replacer could decrease the content of SFA while increasing the UFA contents of formulated sausages or meat batters [5,6,27]. These results suggest that the inclusion of oleogels in the sausage formulations confers healthier characteristics, as the frequent and moderate consumption of oleic acid reduces the risk factors related to the emergence of cardiovascular diseases, such as obesity, hypertension and cholesterol [26]. The ratio of PUFA to SFA (P:S) was 0.25 for the control sample, and it increased from 0.55 to 4.37 when the oleogel fraction increased from 25% to 100%. It has been recommended that the ratio of P:S should be higher than 0.4 for a healthy profile [28]. In addition, the fat replacement by oleogels significantly reduced the index of atherogenicity (IA) and the index of thrombogenicity (IT) of emulsified sausages, which was beneficial for decreasing the risk of coronary artery disease [29].

## 3. Conclusions

Oleogels based on Tween 80 and Arabic gum in combination with different FG concentrations were prepared by an emulsion template method. The higher the FG concentrations, the more stable the O/W emulsion system. Increased concentrations of FG contributed to a more compact gel network structure with stronger mechanical strength as well as a lower oil loss rate. Replacing the pork back fat with oleogels increased the protein contents while decreasing the fat amounts in emulsified sausages. The WHC of emulsified sausages formulated with oleogels as a fat replacer slightly increased. The springiness of emulsified sausages was not affected by the presence of oleogels, and the hardness of emulsified sausages formulated with 100% oleogels was similar to that of the control group. More importantly, the addition of oleogels led to a reduction in SFA content and an increase in UFAs. The nutritional and technological properties of emulsified sausages could be modified by varying the oleogel amount. Overall, the oleogels could be a promising candidate for animal fat in formulated sausages.

## 4. Material and Methods

### 4.1. Materials

Flaxseed gum was provided by Xinjiang Lishide Biotechnology Co., Ltd. (Gongliu, China), which contained 70.92% polysaccharide, 16.20% protein and 12.88% ash. Polysorbate (Tween) 80 was purchased from Shengda Food Additives Co., Ltd.; Arabic gum was purchased from Shanghai Ron Company. Commercial soybean oil was purchased from a local supermarket; Nile red and Nile blue were obtained from Sigma-Aldrich (St. Louis, MO, USA). Pork lean meat and pork fat were purchased from the local market (Tianjin, China). All other chemicals were of analytical grade.

### 4.2. Preparation of Emulsions 

The measure was according to Meng et al. [8] with minor modification. An aqueous solution containing Tween 80 (3.0 wt.%), Arabic gum (0.5 wt.%) and varied concentrations of flaxseed gum (0.1 wt.%–0.9 wt.%) was prepared in deionized water, followed by stirring vigorously at room temperature for 1 h and hydrating overnight at 4 °C. Afterwards, soybean oil (70 wt.%) was mixed with the aqueous solution and sheared at 10,000 rpm for 3 min by using a high-speed homogenizer (IKA T25 digital Ultraturrax, GmbH, Staufen, Germany) to prepare the emulsion. 

### 4.3. Characterization of Emulsions

#### 4.3.1. Droplet Size Distribution

The particle size of the emulsion was determined by a laser diffraction particle size analyzer (LS 13320, Beckman Coulter Ltd., Miami, FL, USA) equipped with a dispersion device. Deionized water was used as the dispersion medium of the liquid. The refractive index of the dispersed phase and that of the dispersed liquid (water) was 1.48 and 1.33, respectively. Each emulsion sample was slowly added to the sample cell and added to 8–12%. The average droplet size in the liquid was described as a volume-weighted average diameter (d_4,3_), and all batches were measured in triplicate. The particle size of the emulsion was manufactured according to a previous approach [12]. 

#### 4.3.2. Microstructural Observation 

The microstructure of the emulsion was observed with a fluorescence inverted microscope (Olympus CKX41, Olympus Corporation, Tokyo, Japan), at a magnification of 40× under a bright field. Before observation, the emulsion droplets were diluted 10 times with distilled water to avoid the accumulation of droplets. The microstructure of the emulsion was manufactured according to a previous approach [12].

#### 4.3.3. Rheological Properties of Emulsions 

The rheological measurement of the emulsion was according to Sun et al. [14]. The rheological measurement of the prepared emulsion was carried out using a rheometer (MARS 60, German Thermal Power Company, Karlsruhe, Germany) equipped with a 35 mm diameter of parallel plate (1 mm gap). The apparent viscosity of the emulsion was measured with a shear rate of 0.01–100 s^−1^ at a constant frequency of 1 Hz. The linear viscoelastic region (LVR) was determined with a strain range of 0.1–100%, at a frequency of 1 Hz. A frequency sweep from 0.1 Hz to 10 Hz at a constant strain of 1% (within the LVR) was performed to obtain the storage modulus (G′) and loss modulus (G″). All measurements were performed using freshly prepared emulsions and oleogels in triplicate at 25 °C.

### 4.4. Preparation and Characterization of Oleogels

#### 4.4.1. Preparation of Oleogels by Emulsion-Templated Approach

The prepared emulsion samples (as described in Section 4.2) were freeze-dried, followed by being sheared with magnetic stirring at 500 rpm for 2 min to obtain the oleogels. 

#### 4.4.2. Microstructural Observation

The morphological structure of the O/W emulsions and oleogels was observed via confocal laser scanning microscopy (CLSM) (Zeiss LSM 980, Oberkochen, Germany). The oil phase was stained with Nile red (0.1%, *w*/*v*, isopropanol solution), and the aqueous phase containing FG was stained with fluorescent white (0.1%, *w*/*v*). The stained samples were stored in a dark place for 1 h. Afterwards, 5 μL of emulsion or a thin slice of oleogel was placed onto a concave slide and observed at 40× magnification. The Nile red was excited with an argon laser at 488 nm, and the fluorescent white was excited with a He/Ne laser at 405 nm.

#### 4.4.3. Viscoelastic Properties 

Rheological measurements of the FG/GA/Tween 80-based oleogels were measured according to the method described in Section 4.3.3. Flow measurement was carried out with shear rates ranging from 0.01 s^−1^ to 100 s^−1^. The frequency sweep test (0.1–10 Hz, strain = 1%) was performed to determine the G′ and G″ of oleogels.

#### 4.4.4. ATR-FTIR

FTIR spectra of formed oleogels were acquired using an IS50 FTIR spectrophotometer (Nicolet IS50, Thermo Fisher Scientific, Madison, WI, USA) equipped with attenuated total reflection (ATR) mode. The spectra were obtained in the range of 400–4000 cm^−1^ at a resolution of 4 cm^−1^ with 32 scans. OMNIC software (Thermo Electron Corp., Madison, WI, USA) was used for analysis.

#### 4.4.5. Oil loss Analysis

The centrifuge method was used to determine the OL values of the oleogel samples [8]. About 2 g of freshly prepared oleogels were placed in a 2.0 mL plastic centrifuge tube, followed by being centrifuged at 10,000 rpm for 15 min (ZONKIA, Hefei, China). Afterwards, the centrifuge tube was inverted for 10 min, and the released oil was removed by filter paper. The oil loss was calculated by the following equation:

(1) $$\mathrm{Oil}\;\mathrm{less}\;(\%)=\frac{M_{2}-M_{1}}{M_{2}}\times 100$$

where *M*_2_ and *M*_1_ indicate the weight of the sample before centrifugation and the weight after oil drainage, respectively.

### 4.5. Preparation of Emulsified Sausage

The whole ingredients employed in the formulations are shown in Table 3. Five batches of different emulsified sausages were designed to contain varying fractions of oleogels as follows: The control group was prepared with 20% pig back fat. The other four groups were formulated by the incorporation of oleogel to achieve the fat replacement. The R-25 group contained 5% oleogel and 15% pig back fat. In R-50 and R-75 samples, 50% and 75% of pig back fat were replaced by the oleogels. In the R-100 sample, pig back fat was totally replaced by the oleogel. The substances in the table were put into the chopping machine, and the emulsified minced meat was obtained after chopping for 30 min. The emulsified minced meat was filled into a plastic shell (diameter 20 mm). The percentage of fat substitution was calculated based on 10 g pig back fat. The amount of ice water added was calculated based on the weight of lean meat and pig back fat. Other ingredients were calculated based on the weight of lean meat, pork back fat and fat substitutes. Emulsified sausages were manufactured according to a previous approach [29].

### 4.6. Proximate Composition and Color Determination

The moisture content of emulsified sausages was determined according to the AOAC method [30]. The fat and protein contents were determined by Soxhlet extraction and the Kjeldahl method, respectively [30]. The color parameters of the samples were measured using a precision colorimeter NR10QC (Shenzhen Threenh Technolgy Co., Ltd., Shenzhen, China). The color was recorded using CIE-L*a*b*. Each experiment was performed at least in triplicate.

### 4.7. Texture Profile Analysis of Emulsified Sausages

Textural parameters were measured using a TA-XT plus/30 analyzer (Stable Micro Systems, Surrey, UK) equipped with a kg load cell, according to previous studies [31] with slight modifications. Before the test, the samples were cut into cylinders with a diameter of 25 mm and a height of 10 mm. Subsequently, the samples were compressed twice with a P/100 probe at 50% strain, and the test speed was 2 mm/s. Texture parameters including hardness, springiness, cohesiveness and chewiness were recorded. For each treatment, three samples were measured and averaged.

### 4.8. Water-Holding Capacity

The WHC of samples was determined following the method of Geng et al. [29] with slight modifications. An amount of 3 g of sample was placed in a centrifuge tube containing skimmed cotton at the bottom and centrifuged at 3000× *g* for 15 min. Each experiment was performed in triplicate. The WHC was obtained by the equation as follows:

WHC(%) = (X_2_ − X_1_)/X_1_ ∗ 100 (2)

where X_1_ denotes the weight of sample before centrifugation, and X_2_ denotes the weight of samples after centrifugation, respectively.

### 4.9. Cooking Loss

Cooking loss of emulsified sausages was determined according to Geng et al. [29] with some modifications. The raw sausages were weighed and then cooked at a temperature of 70 °C for 90 min. After, cooling was performed across three emulsified sausages form each batch. Samples were weighed before and after cooking. The percentage of cooking loss was calculated as follows:

Cooking loss(%) = (*M*_2_ − *M*_1_) / *M*_1_ ∗ 100 (3)

where *M*_2_ presents the weight of sausages before cooking, and *M*_1_ presents the weight of sausages after cooking.

### 4.10. Microstructure of Emulsified Sausage

The micromorphological structure of the sausages was observed by confocal laser scanning microscopy (Zeiss LSM 980, Oberkochen, Germany). Nile red (0.1%, *w*/*v*, isopropanol solution) and fluorescent white (0.1%, *w*/*v*) were used to stain fat and polysaccharide, separately. The emulsified sausage was cut into slices (0.2 cm × 0.2 cm × 0.2 cm) using the razor blade, followed by being stained with 10 μL Nile red and fluorescent white for 1 h. The Nile red and fluorescent white were excited 488 nm and 405 nm.

### 4.11. Fatty Acid Analysis

Extraction of lipids from emulsified sausage samples was performed with normalization solution according to Berker et al. [32]. Afterwards, the lipid phase was saponified and methylated. Fatty acid methyl esters (FAME) were injected into an Agilent7890A gas chromatographer (Agilent Technologies, Palo Alto, CA, USA) that was fitted with a silica capillary column. (HP-88, column length 100 m, inner diameter 0.25 mm, film thickness 0.2 μm). Carrier gas was nitrogen with 0.45–0.5 MPa. The inlet temperature was set to 270 °C, and the detector temperature was 280 °C. The initial temperature of the oven was 100 °C for 13 min. Then, at 100–180 °C, the heating rate was 10 °C/min and kept for 6 min. The heating rate of 180–200 °C was 1 °C/min and kept for 20 min. The heating rate of 200–230 °C was 4 °C/min and kept for 10.5 min. The 1 μL sample was injected into the inlet at a split ratio of 10:1, and the flow rates of hydrogen and air were 40 mL/min and 400 mL/min, respectively. The samples in the experiment were in three copies, and the average value was used in the statistical results.

### 4.12. Statistical Analysis

Each experiment was performed in triplicate, and results were expressed as mean ± SD. The significant difference of data was analyzed by ANOVA and Duncan’s test (*p* < 0.05) in SPSS 27.0 software (SPSS, Inc., Chicago, IL, USA).

## Figures and Tables

**Table 1:** Proximate composition and color values of the emulsified sausage.

	Control	R-25	R-50	R-75	R-100
Moisture(%)	60.15 ± 0.33 ^e^	60.73 ± 0.18 ^d^	61.63 ± 0.31 ^c^	62.74 ± 0.20 ^b^	63.24 ± 0.25 ^a^
Protein(%)	30.25 ± 0.30 ^c^	31.15 ± 0.26 ^ab^	30.80 ± 0.14 ^b^	31.33 ± 0.32 ^a^	30.71 ± 0.26 ^bc^
Fat(%)	28.53 ± 0.24 ^e^	21.55 ± 0.25 ^d^	14.55 ± 0.07 ^c^	7.55 ± 0.12 ^b^	1.52 ± 0.06 ^a^
L*	66.47 ± 0.38 ^d^	68.24 ± 0.40 ^c^	73.39 ± 0.12 ^b^	74.76 ± 0.09 ^a^	75.04 ± 0.18 ^a^
a*	7.27 ± 0.13 ^a^	6.75 ± 0.11 ^b^	6.48 ± 0.10 ^bc^	6.57 ± 0.17 ^bc^	6.52 ± 0.11 ^bc^
b*	12.67 ± 0.41 ^c^	12.50 ± 0.08 ^c^	12.77 ± 0.07 ^bc^	13.39 ± 0.27 ^a^	13.12 ± 0.12 ^ab^
Whiteness	63.42 ± 0.56 ^d^	65.20 ± 0.43 ^c^	69.78 ± 0.11 ^b^	70.68 ± 0.21 ^a^	71.05 ± 0.16 ^a^

Values are displayed by mean ± standard deviation, where different letters represent significant differences (*p*< 0.05).

**Table 2:** Impact of the partial and total replacement of pork back fat by oleogel on fatty acid profile (expressed as g/100 g lipid) of emulsified sausage.

	Control	R-25	R-50	R-75	R-100
C4:0	1.22 ± 0.11 ^a^	1.18 ± 0.17 ^a^	0.95 ± 0.07 ^b^	0.93 ± 0.09 ^b^	0.71 ± 0.04 ^c^
C10:0	0.15 ± 0.04 ^a^	0.12 ± 0.03 ^ab^	0.11 ± 0.01 ^ab^	0.09 ± 0.01 ^b^	0.07 ± 0.01 ^b^
C11:0	0.14 ± 0.03 ^a^	0.13 ± 0.03 ^a^	0.10 ± 0.02 ^ab^	0.09 ± 0.01 ^bc^	0.06 ± 0.01 ^ac^
C13:0	0.09 ± 0.00 ^b^	0.12 ± 0.05 ^b^	0.08 ± 0.01 ^b^	0.22 ± 0.07 ^a^	0.06 ± 0.00 ^b^
C14:0	1.10 ± 0.07 ^a^	0.41 ± 0.09 ^b^	0.27 ± 0.01 ^c^	0.18 ± 0.03 ^cd^	0.13 ± 0.06 ^d^
C16:0	12.11 ± 1.04 ^a^	8.64 ± 0.45 ^b^	5.23 ± 0.52 ^c^	4.31 ± 0.56 ^c^	2.09 ± 0.16 ^d^
C17:0	21.56 ± 4.67 ^b^	29.54 ± 1.91 ^a^	22.74 ± 1.28 ^b^	13.38 ± 0.77 ^c^	8.29 ± 1.40 ^d^
C18:1*n*9t	3.75 ± 0.43 ^a^	2.19 ± 0.21 ^b^	1.58 ± 0.19 ^c^	1.30 ± 0.05 ^c^	0.66 ± 0.14 ^d^
C18:0	14.11 ± 1.85 ^a^	9.43 ± 0.37 ^b^	8.87 ± 0.55 ^b^	5.39 ± 0.65 ^c^	3.70 ± 0.37 ^c^
C18:2*n*6c	5.01 ± 0.86 ^e^	15.97 ± 0.99 ^d^	27.69 ± 1.54 ^c^	38.43 ± 0.88 ^b^	50.26 ± 3.07 ^a^
C20:0	27.19 ± 1.68 ^a^	17.73 ± 1.16 ^b^	13.91 ± 0.80 ^c^	8.96 ± 0.75 ^d^	2.45 ± 0.58 ^d^
C18:3*n*6	9.35 ± 0.38 ^a^	5.44 ± 0.03 ^b^	4.09 ± 0.26 ^c^	3.62 ± 0.30 ^c^	2.42 ± 0.34 ^e^
C18:1*n*9c	2.15 ± 0.37 ^e^	6.84 ± 0.22 ^d^	11.87 ± 0.66 ^c^	20.69 ± 1.47 ^b^	27.42 ± 1.25 ^a^
C22:0	1.16 ± 0.07 ^c^	1.61 ± 0.11 ^c^	2.29 ± 0.18 ^b^	3.16 ± 0.42 ^a^	2.90 ± 0.45 ^a^
C22:6*n*3	0.92 ± 0.04 ^a^	0.65 ± 0.12 ^b^	0.65 ± 0.11 ^b^	0.62 ± 0.05 ^b^	0.33 ± 0.14 ^c^
∑SFA	57.04 ± 2.95 ^a^	39.13 ± 0.79 ^b^	31.64 ± 1.45 ^c^	23.03 ± 0.98 ^d^	12.05 ± 0.88 ^e^
∑MUFA	5.09 ± 0.75 ^e^	9.03 ± 0.51 ^d^	13.44 ± 0.80 ^c^	21.99 ± 0.52 ^b^	28.08 ± 1.21 ^a^
∑PUFA	14.35 ± 1.13 ^e^	21.40 ± 1.02 ^d^	31.78 ± 1.29 ^c^	42.05 ± 1.09 ^b^	52.68 ± 2.74 ^a^
∑UFA	21.17 ± 1.92 ^a^	31.09 ± 1.50 ^b^	45.44 ± 2.41 ^c^	63.30 ± 1.53 ^d^	79.54 ± 2.13 ^e^
PUFA/SFA	0.25 ± 0.01 ^d^	0.55 ± 0.02 ^d^	1.01 ± 0.09 ^c^	1.83 ± 0.12 ^b^	4.37 ± 0.53 ^a^
*n*-6/*n*-3	0.53 ± 0.08 ^d^	2.94 ± 0.17 ^d^	6.76 ± 0.81 ^c^	10.61 ± 0.79 ^b^	20.80 ± 4.29 ^a^
IA	1.51 ± 0.01 ^a^	0.65 ± 0.05 ^b^	0.34 ± 0.04 ^c^	0.16 ± 0.01 ^d^	0.08 ± 0.00 ^e^
IT	0.57 ± 0.02 ^a^	0.29 ± 0.02 ^b^	0.16 ± 0.02 ^c^	0.08 ± 0.00 ^d^	0.04 ± 0.00 ^e^

SFA = saturated fatty acids, MUFA = monounsaturated fatty acids, PUFA = polyunsaturated fatty acids, *n*-6 = omega-6; *n*-3 = omega-3. IA(Index of atherogenicity) = (4 × C14:0 + C16:0 + C18:0)/(∑MUFA + ∑PUFA-*n*6 + ∑PUFA-*n*3). IT(Index of thrombogenicity) = (C14:0 + C16:0 + C18:0)/(0.5 × ∑MUFA + 0.5*∑PUFA-*n*6 + 3∑PUFA-*n*3 + ∑PUFA-*n*3/∑PUFA-*n*6). The lowercase letters indicated the significance between samples.

**Table 3:** Formulations of the emulsified sausage.

	Control	R-25	R-50	R-75	R-100
Lean meat	40 g	40 g	40 g	40 g	40 g
Pork back fat	10 g	7.5 g	5 g	2.5 g	0
Fat replacer	0 g	2.5 g	5 g	7.5 g	10 g
Ice water	15 g	14.25 g	13.5 g	12.75 g	12 g

All samples also contain: 5% starch, 1.6% salt, 6% granulated sugar, 0.4% composite phosphate, 0.14% aginomoto, 0.04% five-spice powder, 0.04% ginger powder and 0.08% monascus red.

## Data Availability

Not applicable.
